# Possible protective role of the absence of Hyrtl’s anastomosis in monochorionic pregnancy: exploratory case series

**DOI:** 10.3389/fmed.2025.1575068

**Published:** 2025-05-30

**Authors:** Ziling Liu, Jie Ruan

**Affiliations:** Key Laboratory of Birth Defects and Related Diseases in Women and Children, Department of Obstetrics and Gynecology, Ministry of Education, West China Second Hospital, Sichuan University, Chengdu, China

**Keywords:** Hyrtl’s anastomosis, monochorionic pregnancy, placental territory discordance, selective fetal growth restriction, pump fetus

## Abstract

**Objective:**

Hyrtl’s anastomosis, an intra-arterial shunt, serves a protective function in singleton placentas. Our team previously documented a case of a monochorionic (MC) twin placenta lacking Hyrtl’s anastomosis. However, research on the impact of absent Hyrtl’s anastomosis in twin placentas remains limited.

**Methods:**

In the present study, we perfused over 70 cases of MC placentas and identified three additional cases exhibiting the same characteristics.

**Results:**

The absence of Hyrtl’s anastomosis was found to be present in two cases with severe placental territory discordance and one case of a MC triamniotic pregnancy with reversed arterial perfusion sequence, all of which resulted in uneventful pregnancy courses.

**Conclusion:**

The absence of Hyrtl’s anastomosis appears to have a protective effect in MC placentas, contrary to its effect in singleton placentas.

## Objective

Hyrtl’s anastomosis describes a connection between the two umbilical arteries before they insert into the placenta (1). Previous studies have found that Hyrtl’s anastomosis provides a protective effect on singleton placentas by equalizing pressure between the arteries, acting as a safety valve in certain situations like differences in the size of areas supplied by the arteries, placental compression, or blockage of one artery (1, 2).

However, research on Hyrtl’s anastomosis in twin placentas is limited. Our team previously reported a case of a monochorionic diamniotic (MCDA) twin placenta lacking Hyrtl’s anastomosis, concluding that its effects differ in MC placentas compared to singleton placentas (3). To our knowledge, only one other similar case has been reported (4). To validate our findings, we perfused over 70 cases of MC placentas and identified three additional cases with the same characteristics, further supporting that the absence of Hyrtl’s anastomosis may play a beneficial role in MC twins.

## Methods

Over the past 2 years, we have perfused more than 70 cases of MC placentas using acrylic dyes. Following flushing of the placental vessels, all intact MC placentas were injected with dye to examine superficial anastomoses, placental territory, and umbilical cord insertion of the twins. Digital images were stored for the following data analysis. The individual placental territory was marked by ImageJ software as follows: on the image of the chorionic fetal surface of the placenta, the vascular equator was delineated by connecting the avascular zone and all visible anastomoses from one edge of the placenta to the other, thereby dividing the placenta into two territories. Individual placental territory was calculated as follows: (individual placental area/total placental area) × 100. The absence of Hyrtl’s anastomosis was confirmed in four MC placentas, including the first case previously published (3). Below is the information on three new cases, with additional detailed ultrasound findings presented in Supplementary Table S1.

## Results

### Case 1

**Figure 1:**
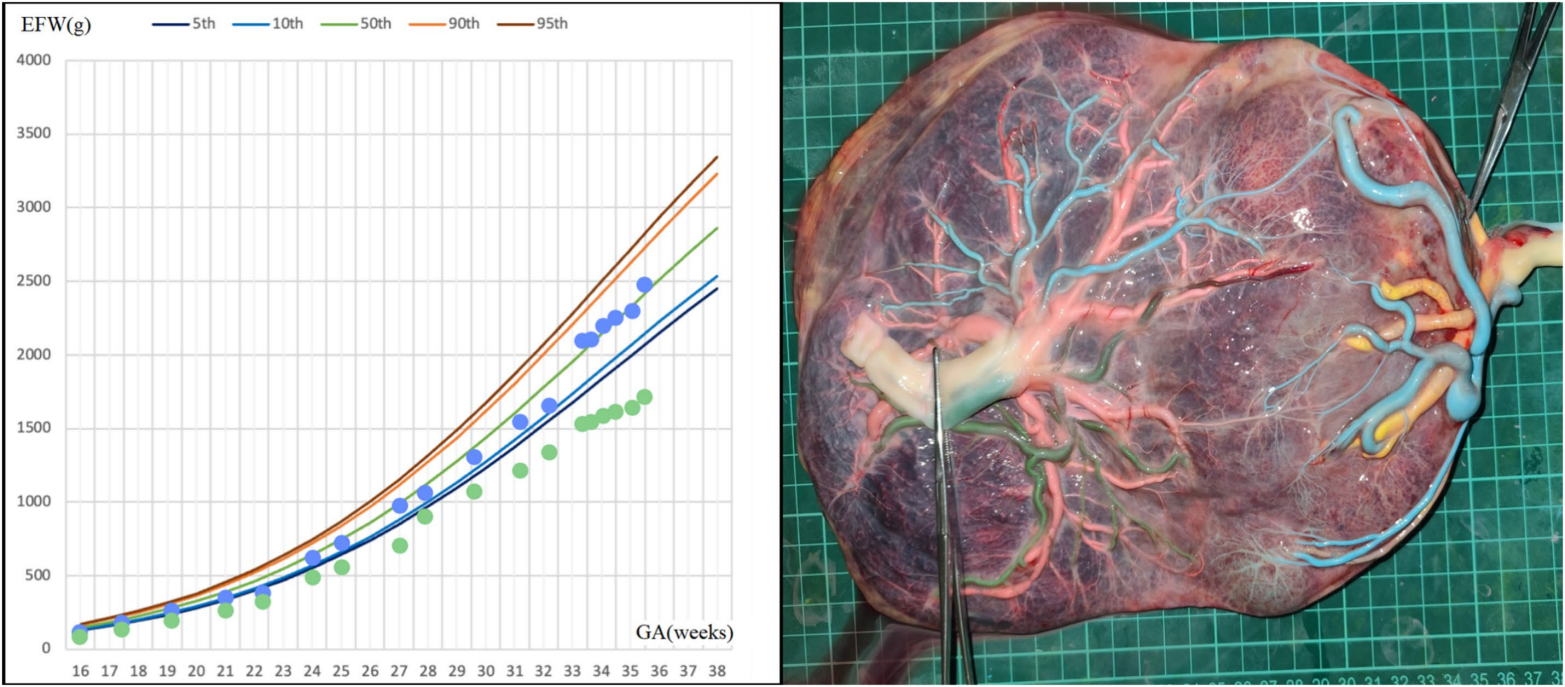
Fetal growth velocity in two fetuses and placental perfusion staining of case 1. (Left: the blue dots represent EFW of the larger fetus at different weeks of gestation, and the green dots represent EFW of the smaller fetus at different weeks of gestation. Right: the umbilical vein of the larger fetus was perfused with pink dye, and the umbilical vein of the smaller fetus was perfused with yellow dye. Umbilical arteries of the smaller fetus were perfused with blue dye, and blue staining of the branch vessels of the two umbilical arteries of the smaller fetus and one umbilical artery of the larger fetus through the arterial–arterial anastomosis was observed. The other umbilical artery of the larger fetus was perfused with green dye. No color mixing was observed between the two umbilical arteries of the larger fetus and their branch vessels). ^*^GA, gestational age; EFW, estimated fetal weight.

A 31-year-old woman conceived twins through *in vitro* fertilization (IVF). An ultrasound examination at 12 weeks confirmed a MCDA twin pregnancy. At 15 + 5 weeks, there was a significant estimated fetal weight difference between the twins, with positive diastolic flow in the umbilical artery (UA) of the small twin. An invasive test for fetal karyotyping and chromosomal microarray analysis (CMA) was conducted to rule out chromosomal abnormalities in both fetuses. At 22 weeks, UA Doppler flow abnormalities were observed in the smaller fetus (UA systolic/diastolic ratio = 6.54, UA pulsatility index = 1.69), and UA absent end-diastolic velocity (AEDV) was shown on a repeat ultrasound examination 1 week later, leading to a diagnosis of type III selective fetal growth restriction (SFGR). However, weekly monitoring from 24 weeks showed positive diastolic flow in the UA in both fetuses; amniotic fluid amount, the middle cerebral artery (MCA) peak systolic velocity, and ductus venosus (DV) Doppler were all also shown to be normal. At 34 weeks, an elective cesarean section was recommended but declined by the mother. However, the growth rate of the smaller fetus began to slow (Figure 1), prompting an elective cesarean section at 35 + 5 weeks. The twins were born with birth weights of 2,410/1,660 grams, showing a birth weight discordance ratio of 31%, and both had a 1-min Apgar score of 10. The postpartum course was uneventful for both the mother and the larger infant. The smaller infant was hospitalized in the neonatal intensive care unit (NICU) for 11 days, receiving phototherapy for neonatal jaundice, and was discharged upon reaching a weight of 1,800 g with normal follow-up. Placental perfusion staining indicated a placental territory discordance ratio of 64.3%, with marginal cord insertion for the smaller fetus. Additionally, there was no Hyrtl’s anastomosis between the two umbilical arteries of the larger fetus (Figure 1).

### Case 2

**Figure 2:**
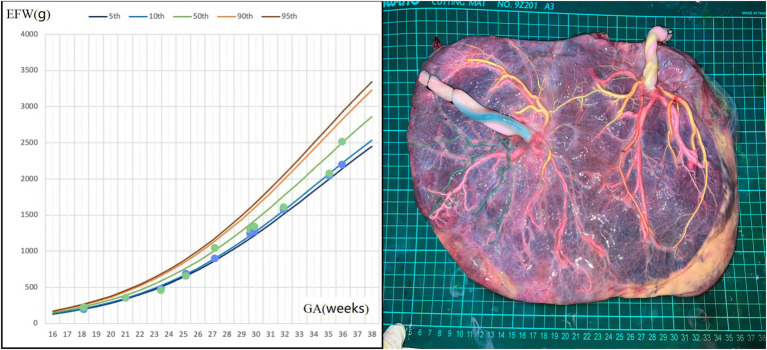
Fetal growth velocity in two fetuses and placental perfusion staining of case 2. (Left: the blue dots and green dots represent EFW of the two fetuses at different weeks of gestation. It can be seen that the weight gain of the twins was very close, despite a large placental territory discordance. Right: the umbilical vein of the two fetuses were perfused with pink dye. Umbilical arteries of the fetus with a smaller individual placental area were perfused with yellow dye, and yellow staining of the branch vessels of the two umbilical arteries of this fetus and one umbilical artery of another fetus through the arterial–arterial anastomosis was observed. The other umbilical artery of the other fetus was perfused with green dye. No color mixing was observed between the two umbilical arteries of the fetus with a larger individual placental area). ^*^GA, gestational age; EFW, estimated fetal weight.

A 37-year-old woman, with a history of a spontaneous miscarriage at 24 weeks, conceived through IVF. Early pregnancy ultrasound examination indicated MCDA twin fetuses, and her non-invasive prenatal test (NIPT) showed low risk. During mid-pregnancy, she was diagnosed with gestational diabetes, which was well-controlled. Regular ultrasound examination showed normal levels of amniotic fluid and normal blood flow in UA, MCA, and DV in both fetuses. However, the estimated weight of one fetus fluctuated between the 8th and 20th percentiles of Asian fetuses at the same gestational age (Figure 2). At 36 + 5 weeks, the patient presented with vaginal discharge and was suspected to have premature rupture of the membranes. An emergency cesarean section was performed, delivering two fetuses weighing 2,180/2,330 grams, both with Apgar scores of 10 at 1 min. The postpartum course was uneventful for both the mother and the larger infant, while the smaller infant was transferred to the NICU for 4 days and received phototherapy. Placental perfusion staining indicated a placental territory discordance ratio of 44.6%. Additionally, the fetus with a greater placental area and birth weight lacked Hyrtl’s anastomosis between the two umbilical arteries (Figure 2).

### Case 3

**Figure 3:**
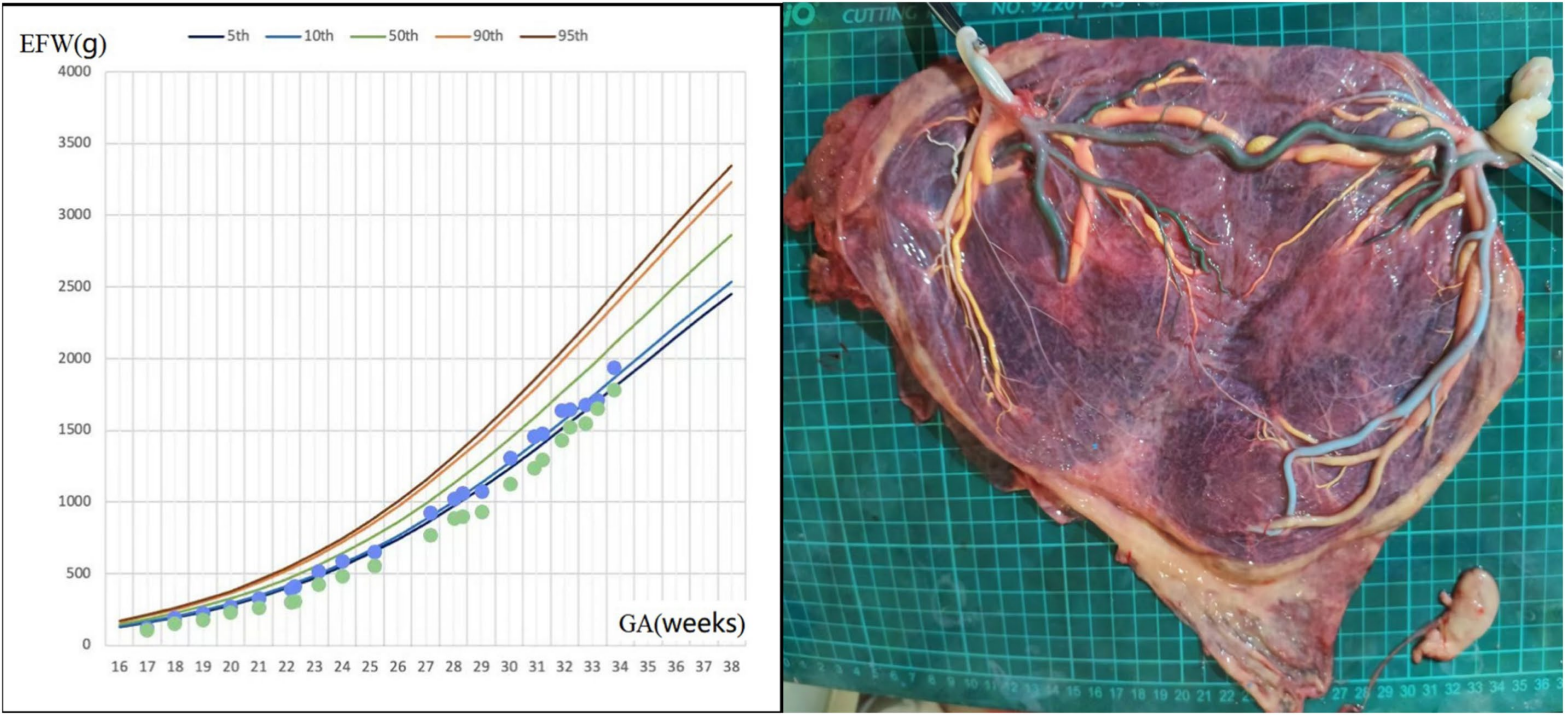
Fetal growth velocity in two fetuses and placental perfusion staining of case 3. (Left: the blue and green dots represent EFW of the two fetuses at different weeks of gestation. Right: the umbilical vein of two fetuses were perfused with yellow dye, and large veno-venous anastomosis was seen. One umbilical artery of one pump fetus was perfused with green dye, and green staining of the branch vessels of one umbilical artery of the other pump fetus through the arterial–arterial anastomosis was observed. The other umbilical artery of the two pump fetuses were perfused with blue dye and white dye, respectively. No color mixing was observed between the two umbilical arteries of two pump fetuses. The vessels of the acardiac fetus had atrophied). ^*^GA, gestational age; EFW, estimated fetal weight.

A 33-year-old woman, gravida 1 para 0, spontaneously conceived. An ultrasound examination at 12 gestational weeks confirmed monochorionic triamniotic (MCTA) triplets, including one acardiac fetus (fetus 1) and two normal fetuses (fetus 2 and fetus 3). She had a previous diagnosis of chronic nephritis, but her urine protein and serum creatinine levels remained stable during pregnancy. At 14 weeks, ultrasound examination showed the acardiac fetus measuring 5.2 × 3.1 × 3.3 cm (estimated weight of 28 grams), while fetuses 2 and 3 were estimated to weigh 81/68 grams, respectively, so fetal reduction was not considered. Amniocentesis for fetuses 2 and 3 revealed no chromosomal abnormalities. Serial ultrasound examinations initiated at 16 weeks of gestation revealed severe growth restriction in fetus 3, associated with intermittent AEDV in UA Doppler. Concurrently, the acardiac twin exhibited no significant volumetric increase, and its umbilical artery (UA) blood flow progressively diminished to undetectable levels. The growth curve of fetuses 2 and 3 was shown in Figure 3. Fetal cardiac ultrasound at 24 weeks indicated that fetus 3 had a permanent left superior vena cava. At 33 weeks, the patient was admitted for closer monitoring, and an elective cesarean section was performed at 34 weeks. The birth weights of fetuses 2 and 3 were 1,910/1,370 grams, respectively, and both had Apgar scores of 9 and 10 at 1 and 5 min. Both twins were admitted to the NICU due to low birth weight. They received non-invasive ventilatory support and phototherapy and were discharged after 13 days of hospitalization upon reaching weights of 2,060/1,880 grams, respectively, with no significant abnormalities on follow-up. Placental perfusion staining indicated a placental territory discordance ratio of 12.7%, with marginal cord insertion for the smaller fetus. Additionally, there was a lack of Hyrtl’s anastomosis between the two umbilical arteries in both live fetuses (Figure 3).

## Discussion and conclusions

Although all guidelines recommended the perfusion of MC placentas, in practice, few obstetricians actually perform this procedure for various reasons. Furthermore, the limited larger-sample studies on MC placental perfusion predominantly focus on pathological conditions such as SFGR, twin–twin transfusion syndrome (TTTS), and twin anemia polycythemia sequence (TAPS). Consequently, there is limited interest among obstetricians in the vascular distribution characteristics of uncomplicated MC placentas.

Multiple previous studies have concluded that arterial–arterial (AA) anastomosis exerts a protective effect on MC twins by allowing rapid bidirectional blood flow at the site, thereby compensating for blood volume imbalances between the twins (5). Other reported protective factors include concordance in umbilical cord insertion sites and placental territory (6, 7). Our three cases demonstrate that the spatial distribution of blood vessels in the MC placenta forms an intricate three-dimensional structure, suggesting the presence of additional protective factors that may help balance the two fetuses.

Walker et al. (4) reported a case in which the absence of Hyrtl’s anastomosis in the umbilical cord of the donor twin delayed the deterioration of TTTS. The authors postulate that the absence of Hyrtl’s anastomosis effectively isolated the circulations of the two umbilical arteries, thereby preventing excessive blood flow from the donor to the recipient.

Our team identified four similar cases. In our previously published case, despite a significant difference in placental volume, the birth weight difference between the twins was not substantial (3). Initially, we believed the difference between our case and the case of Liu and Ruan (3) was that the absent Hyrtl’s anastomosis occurred in the larger twin rather than the smaller twin. However, in cases of SFGR, the larger twin provides blood support to the smaller twin via an AA anastomosis to compensate for the relatively smaller placental volume, thereby functioning as the donor. Thus, the essence of our first case is similar to that of Martin et al., where the absence of Hyrtl’s anastomosis effectively isolates the circulations of the two umbilical arteries, preventing excessive blood loss from the donor to the recipient.

The three newly identified cases in this study exhibited similar characteristics. In case 1 and 2, the fetuses with relatively larger placental volume served as the donor, while in case 3, both fetus 2 and fetus 3 were pump fetuses. In all three cases, absent Hyrtl’s anastomosis was observed in donor fetuses, yet the pregnancy courses were uneventful. Our findings indicate that the absence of Hyrtl’s anastomosis permits relatively independent placental circulations in donors of MC multiple pregnancies, allowing the recipient fetus to be adequately compensated while ensuring sufficient cotyledonary perfusion and oxygenation for the donor.

Our study is based on postpartum placental anatomical findings. In routine clinical practice, prenatal ultrasonographic measurement of Hyrtl’s anastomosis is rarely performed. However, the application of prenatal ultrasound for assessing Hyrtl’s anastomosis is feasible. Raio et al. (2) conducted measurements of Hyrtl’s anastomosis, including anastomotic diameter and Doppler flow velocity waveforms, in 41 women with singleton pregnancies in the third trimester. In the study by Qu et al. (8), 2D time-of-flight magnetic resonance angiography successfully demonstrated Hyrtl’s anastomosis in two pregnant women. Therefore, we hope this study could be extended prospectively, with additional prenatal imaging assessments of Hyrtl’s anastomosis in MC twins, to determine whether its absence carries clinical significance. Nevertheless, it must be acknowledged that some Hyrtl’s anastomoses might be misclassified as absent prenatally due to observational challenges.

In conclusion, our preliminary observation supports our previous assertion that the absence of Hyrtl’s anastomosis may have a possible protective effect in MC placentas, which contrasts with its effect in singleton placentas. Further cases of placental perfusion staining are needed to reinforce our findings.

## Data Availability

The original contributions presented in the study are included in the article/Supplementary material, further inquiries can be directed to the corresponding author.
