# Supplementary material for: Possible protective role of the absence of Hyrtl’s anastomosis in monochorionic pregnancy: exploratory case series
Source: Front Med (Lausanne). 2025 May 30;12:1575068. doi: 10.3389/fmed.2025.1575068 (PMC12162271; doi:10.3389/fmed.2025.1575068)
Supplement: Supplementary file 1 [file Table_1.docx]

Table S1. Detailed ultrasound findings of 4 cases.

| GESTATIONAL  WEEKS | Case1 |  | Case2 |  | Case3 |  | Case of Reference 3 |  |
| --- | --- | --- | --- | --- | --- | --- | --- | --- |
|  | UA S/D ratio(UAPI) | | | | | | | |
| 16-18 | 6.20(NA) | 4.10(NA) | 4.10(NA) | 4.90(NA) | 3.81(NA) | AEDV | NA | NA |
| 18-20 | 6.58(1.58) | 4.63(1.45) | 4.40(NA) | 4.60(NA) | 5.49(1.45) | 8.69(1.67) | NA | NA |
| 20-22 | 6.54(1.68) | 3.40(1.17) | 3.30(NA) | 4.30(NA) | 4.62(1.40) | AEDV | NA | NA |
| 22-24 | AEDV | 3.92(1.24) | 3.20(NA) | 3.20(NA) | 2.96(1.09) | AEDV | NA | NA |
| 24-26 | 4.35(1.35) | 3.51(1.11) | 3.10(NA) | 3.00(NA) | 2.63(0.95) | iAEDV | 3.16(1.04) | 3.35(1.07) |
| 26-28 | 3.83(1.21) | 3.21(1.04) | 4.70(NA) | 3.70(NA) | 2.10(0.81) | iAEDV | 3.23(1.06) | 4.38(1.50) |
| 28-30 | 2.43(0.85) | 2.47(0.84) | 3.56(1.17) | 2.78(1.02) | 2.90(1.00) | AEDV | 2.23(0.81) | 2.17(0.80) |
| 30-32 | 2.96(0.95) | 2.76(0.91) | 2.80(NA) | 2.90(NA) | 2.88(0.98) | iAEDV | 2.80(1.01) | 3.69(1.19) |
| 32-34 | 2.59(0.99) | 1.67(0.85) | 2.01(0.70) | 2.27(0.81) | 2.93(1.01) | 1.43 | 2.83(1.01) | 2.59(0.87) |
| 34-36 | 2.87(1.07) | 2.38(0.87) | 2.90(1.05) | 2.91(1.02) |  |  | 2.34(0.85) | 2.85(0.99) |
| 36-37 |  |  | 2.07(0.73) | 3.55(1.21) |  |  |  |  |
| Amniotic Fluid Depth(cm) | | | | | | | | |
| 16-18 | 3.6 | 4.3 | 3.4 | 3.3 | 5.6 | 3.9 | NA | NA |
| 18-20 | 3.4 | 4.7 | 5.7 | 4.4 | 6.3 | 4.6 | NA | NA |
| 20-22 | 3.8 | 4.2 | 4.2 | 4.3 | 5.2 | 4.6 | NA | NA |
| 22-24 | 4.6 | 6.0 | 5.3 | 6.2 | 6.6 | 5.0 | NA | NA |
| 24-26 | 5.1 | 6.0 | 4.5 | 3.5 | 7.3 | 4.8 | 5.1 | 5.0 |
| 26-28 | 5.4 | 6.2 | 4.6 | 5.0 | 7.6 | 5.9 | 5.6 | 5.6 |
| 28-30 | 6 | 5.8 | 4.6 | 5.0 | 5.7 | 5.8 | 5.2 | 5 |
| 30-32 | 6.8 | 6.1 | 4.5 | 5.6 | 6.4 | 5.4 | 6.5 | 7.3 |
| 32-34 | 5.8 | 6.2 | 3.5 | 4.3 | 5.2 | 9.2 | 5.3 | 4.7 |
| 34-36 | 3.8 | 3.8 | 4.3 | 4.8 |  |  | 4.8 | 4.4 |
| 36-37 |  |  | 4.9 | 3.2 |  |  |  |  |

UA: umbilical artery; S/D: systolic and diastolic velocity ratio; UAPI: umbilical artery pulsatility index; NA: not applicable; AEDV: absent end-diastolic flow; iAEDV: intermittent absent end-diastolic flow.
